# Impact of Anesthetic Variation in Endovascular Treatment of Acute Ischemic Stroke

**DOI:** 10.7759/cureus.11328

**Published:** 2020-11-04

**Authors:** Arsalaan Salehani, Dwight Self, Bonita Agee, Karim Refaey, Galal A Elsayed, Gustavo Chagoya, Joshua Bernstock, William Stetler

**Affiliations:** 1 Neurological Surgery, University of Alabama at Birmingham, Birmingham, USA; 2 Neurological Surgery, Thomas Jefferson University Hospital, Philadelphia, USA; 3 Neurological Surgery, Mayo Clinic, Jacksonville, USA; 4 Neurological Surgery, Brigham and Women's Hospital, Harvard Medical School, Boston, USA; 5 Neurological Surgery, Carolina Neurosurgery and Spine Associates, Charlotte, USA

**Keywords:** infarction, thrombectomy, anesthesia, sedation, outcomes

## Abstract

Background

Given recent technological advancements leading to better outcomes in endovascular therapy for acute ischemic stroke (AIS), updated guidelines recommend thrombectomy as the standard of care in acute large vessel occlusions. However, use of general anesthesia versus conscious sedation continues to be discussed. Two previous randomized trials have shown no significant difference between the use of conscious sedation compared with general anesthesia.

Methods

The authors performed a retrospective analysis of all consecutive patients with acute ischemia who underwent intra-arterial thrombectomy between September 2014 and May 2020 at a Level 1 stroke center. Patient characteristics along with clinical and operative data were extracted. Frequency distributions of selected characteristics were obtained and statistical significance of any differences according to the mode of anesthesia was assessed.

Results

A total of 480 patients were included in this study, 257 underwent general anesthesia and 223 underwent conscious sedation. Length of stay (LOS) in the ICU nor length of hospital stay was significantly different between groups. Change in National Institutes of Health Stroke Scale (NIHSS) score from admission to discharge, procedure times, and discharge disposition were not found to be significantly associated with either group although there was a trend towards longer door to puncture time with general anesthesia. Discharge disposition was found to be significantly associated with admission NIHSS (p=0.04). There was a trend towards longer hospital stay in patients with worse admission NIHSS (p=0.09). Success of thrombectomy was not significantly different between both anesthesia groups (p=0.37).

Conclusions

This large, single-center retrospective cohort study echoes the results of two previous randomized controlled trials in demonstrating non-inferiority of general anesthesia versus conscious sedation in cases of intra-arterial thrombectomy for AIS. These results contrast those of previously published retrospective studies.

## Introduction

Acute ischemic stroke (AIS) is one of the leading causes of morbidity in the United States [1]. Following recent technological advancements, higher recanalization rates have been achieved with endovascular intra-arterial thrombectomy which is associated with improved functional outcomes and higher rates of patient survival [2,3]. Recent randomized clinical trials, as well as meta-analyses, have shown reduced costs, increased quality of life, increased disability-free survival, and increased life expectancy of patients undergoing thrombectomy combined with maximal medical therapy when compared to maximal medical therapy alone in the management of AIS. This has led to establishing endovascular thrombectomy as the standard of care for patients with AIS secondary to acute large vessel occlusions [4-9].

Given the adoption of thrombectomy as the new standard of care, choice of sedation, and modes of airway management during endovascular therapy have become a topic of great interest [10]. There are a variety of studies, from retrospective analyses to clinical trials, that have shown that general anesthesia leads to worse clinical outcomes such as increased respiratory complications, decreased reperfusion rates, worse functional outcomes, higher hemodynamic instability, higher ventilator-associated complications, and increased procedure time when compared with conscious sedation [7,11-21].

On the other hand, the Sedation versus Intubation for Endovascular Stroke Treatment (SIESTA) trial was the first randomized trial comparing conscious sedation versus intubation in thrombectomy. The SIESTA trial failed to demonstrate superiority of conscious sedation over general anesthesia [22]. Subsequently, the Anesthesia during Stroke (AnStroke) randomized trial also tried to delineate superiority between these two sedation modes. The AnStroke trial found that if profound hypotension did not occur, general anesthesia was not inferior to conscious sedation in thrombectomy therapy [23]. Due to the high diversity in the literature, the aim of this single center study was to analyze the outcomes in patients undergoing general anesthesia compared to those subjected to conscious sedation during endovascular therapy for AIS.

## Materials and methods

Institutional review board approval was obtained for this study. A retrospective chart review was completed for all consecutive patients with acute cerebral ischemia over the age of 18 and who underwent intra-arterial thrombectomy between September 2014 and May 2020 at the University of Alabama at Birmingham. In this series, the vast majority of thrombectomies were performed using the Solumbra technique. The criteria for thrombectomy was Alberta Stroke Program Early CT Score (ASPECTS) and time since last known normal along with clinical evaluation by neurointerventional faculty [24].

Data extraction

Data on age, sex, race, mortality, and comorbidities (e.g. tobacco use, atrial fibrillation, diabetes, hypertension, hyperlipidemia, and coronary artery disease) were collected. Clinical and operative characteristics such as arrival time, blood pressure on arrival, National Institutes of Health Stroke Scale (NIHSS) at admission and discharge (when available), door to puncture time, recanalization time, blood pressure during thrombectomy, thrombolysis in cerebral infarction (TICI) score, and thrombectomy end time. Mode of anesthesia was also recorded (i.e. general anesthesia versus conscious sedation). Subsequent intensive care unit (ICU) and hospital length of stay (LOS) and were recorded along with discharge disposition.

Statistical analysis

Frequency distributions of demographics, comorbidities, length of stay and other selected characteristics were obtained and statistical significance of any differences according to mode of anesthesia was assessed. For categorical variables including gender, race, comorbidities, smoking and intervention chi-square test and Fisher’s exact test, where appropriate, were used. Independent t-test, Wilcoxon rank sums, and median two-sample test were utilized to assess the significance of differences in age, length of stay, time from puncture to recanalization and other continuous variables. Analysis of variance (ANOVA) was used to determine the statistical significance of mean differences in NIHSS scores according to categories of stroke severity. Statistical significance was defined as p < 0.05. All statistical analyses were performed using SAS version 9.4 (SAS Institute, Inc., Cary, NC).

## Results

A total of 480 patients were included in this study, 257 underwent general anesthesia (GA) and 223 underwent conscious sedation (CS). Of these 480 patients, 50.8% (n=244) were male and 49.2% (n=236) were female (Table 1). The majority of patients were Caucasian (64.0%, n=307), with a smaller portion being African American (29.6%, n=142) and Asian (6.4%, n=31). The mean age for patients in this study was 65.2 ± 12.7 (Table 1). Comorbidities such as smoking, atrial fibrillation, diabetes, intra-procedure hypertension (defined as systolic blood pressure > 200), hyperlipidemia, and coronary artery disease were not significantly different between the two groups (Table 1). Intra-procedure hypotension (defined as systolic blood pressure < 90) was more common in the conscious sedation group and approached significance (p=0.09).

**Table 1:** Patient demographics and selected characteristics ICU: intensive care unit

Variable	Total (N=480)	General Anesthesia (n=257)	Conscious Sedation (n=223)	p value*
Gender, n (%)				0.70
Male	244 (50.8)	136 (52.9)	108 (48.4)	
Female	236 (49.2)	121 (47.1)	115 (51.6)	
Race, n (%)				0.56
White	307 (64.0)	167 (65.0)	140 (62.8)	
Black	142 (29.6)	75 (29.2)	67 (30.0)	
Asian	31 (6.4)	15 (5.8)	16 (7.2)	
Age in years, mean ± SD	65.2 ± 12.7	62.7 ± 17.5	63.5 ± 13.3	0.45
Age in years, median (range)	66.0 (23.0-96.0)	63.0 (27.0-96.0)	64.0 (23.0-94.0)	0.68
ICU Length of stay in days, mean ± SD	5.7 ± 6.3	5.4 ± 7.3	5.8 ± 6.5	0.75
ICU Length of stay in days, median (range)	3.0 (0.0-48.0)	4.0 (1.0-25.0)	3.0 (0.0-48.0)	0.87
Hospital length of stay, mean ± SD	9.7 ± 10.1	7.9 ± 10.7	9.4 ± 9.5	0.15
Hospital length of stay in days, median (range)	7.0 (0.0-60.0)	6.0 (1.0-40.0)	7.0 (0.0-60.0)	0.35
Smoking, n (%)	222 (46.3)	105 (40.9)	117 (52.5)	0.57
Atrial fibrillation, n (%)	107 (22.3)	59 (23.0)	48 (21.5)	0.13
Diabetes, n (%)	86 (17.9)	41 (16.0)	45 (20.2)	0.33
Hypertension, (%)	262 (54.6)	133 (51.8)	129 (57.8)	0.21
Hypotension, n (%)	54 (11.3)	23 (8.9)	31 (13.9)	0.09
Hyperlipidemia, n (%)	181 (37.7)	98 (38.1)	83 (37.2)	0.81
Coronary artery disease, n (%)	131 (27.3)	72 (28.0)	59 (26.5)	0.55
Deceased, n (%)	161 (33.5)	82 (31.9)	79 (35.4)	0.62
*p-value statistically significant at p <0.05				
Difference in means analyzed using Wilcoxon Rank Sum test; difference in medians analyzed using				
Median 2-Sample test				

One-hundred sixty-one patients (33.5%) were deceased, with no significant difference between modes of anesthesia (p=0.62) (Table 1). Neither mean nor median ICU or hospital LOS was found to be significantly different between the two sedation groups (Table 1). Two-hundred and fifty-six total patients had NIHSS recorded at both admission and discharge (Table 2). Neither mean (p=0.27) nor median (p=0.52) change in NIHSS from admission to discharge was found to be significantly different in either anesthesia group (Table 2).

**Table 2:** Change in NIHSS from admission to discharge NIHSS: National Institutes of Health Stroke Score

Variable	Total (N=256)	General Anesthesia (n=155)	Conscious Sedation (n=101)	p value*
Change in NIHSS score, mean ± SD	10.5 ± 5.1	11.7 ± 1.7	10.3 ± 4.6	0.27
Change in NIHSS score, median (range)	10.0 (-5.0 – 19.0)	11 (-3.0 – 15.0)	10.0 (-5.0 – 19.0)	0.52
*p-value statistically significant at p <0.05				
Difference in means analyzed using Wilcoxon Rank Sum test; difference in medians analyzed using				
Median 2-Sample test				

Mean door to puncture time was longer in the GA group and approached statistical significance (p=0.07). However, median door to puncture time was not significantly different (p=0.35). Mean and median puncture to recanalization time were not significantly different between both groups (p=0.85 and p=0.63, respectively). Mean total length of thrombectomy case was 1:14:45 ± 0:31:29 and median total length of thrombectomy case was 1:07:00 (0:37:00-2:57:39) for all patients and neither was significantly different between both anesthesia groups (Table 3).

**Table 3:** General anesthesia vs conscious sedation: thrombectomy procedure characteristics

Variable	Total (N=480)	General Anesthesia (n=257)	Conscious Sedation (n=223)	p value*
Door to puncture time,				
Mean ± SD	1:02:17 ± 0:23:09	1:05:45 ± 0:35:43	0:59:35 ± 0:17:45	0.07
Door to puncture time,				
Median (range)	1:01:33 (0:15:00-2:35:08)	1:09:15 (0:26:00-2:35:08)	1:04:17 (0:15:00-1:45:23)	0.35
Puncture to recanalization time,				
Mean ± SD	0:47:47 ± 0:22:34	0:48:51 ± 0:29:58	0:47:34 ± 0:19:27	0.85
Puncture to recanalization time,				
Median (range)	0:46:00 (0:03:00-2:19:00)	0:45:00 (0:03:00-1:57:00)	0:46:00 (0:05:00-2:19:00)	0.63
Total length of thrombectomy case,				
Mean ± SD	1:14:45 ± 0:31:29	1:15:46 ± 0:39:37	1:13:27 ± 0:29:51	0.23
Total length of thrombectomy case,				
Median (range)	1:07:00 (0:37:00-2:57:39)	1:07:30 (0:45:00-2:57:39)	1:05:00 (0:37:00-2:12:49)	0.51
*p-value statistically significant at p <0.05; Times in HH:MIN:SEC format				

One-hundred sixty-five patients (34.4%) were discharged home, 161 patients (33.5%) expired at the hospital, 91 patients (19.0%) were discharged to inpatient rehabilitation (IPR), and 63 patients (13.1%) were discharged to a long-term care or hospice facility (Table 4). Patient discharge location following hospital stay was not found to be significantly associated with either mode of anesthesia (p=0.88) (Table 4). However, disposition was found to be significantly associated with admission NIHSS with the moderate-to-severe and severe admission NIHSS groups having higher numbers of patients expiring in the hospital and the mild and moderate admission NIHSS groups having higher numbers discharging home (p=0.04, Table 5). A trend towards longer hospital LOS was also found in patients with worse admission NIHSS, however, this was not statistically significant (p=0.09, Table 6). Lastly, success of thrombectomy (as represented by TICI score) was not found to be significantly different when comparing the two anesthesia groups (p=0.37, Table 7).

**Table 4:** Disposition after hospital discharge IPR, inpatient rehabilitation

Variable	Total (N=480)	General Anesthesia (n=257)	Conscious Sedation (n=223)	p value*
Disposition, n (%)				0.63
Home	165 (34.4)	85 (33.1)	80 (35.9)	
Expired	161 (33.5)	82 (31.9)	79 (35.4)	
IPR	91 (19.0)	50 (19.5)	41 (18.4)	
Long-term Care/Hospice	63 (13.1)	40 (15.5)	23 (10.3)	
*p-value statistically significant at p <0.05				

**Table 5:** Discharge disposition based on admission NIHSS NIHSS: National Institutes of Health Stroke Score; IPR: inpatient rehabilitation

Variable	Total (N=480)	Mild (n=65)	Moderate (n=114)	Moderate to Severe (n=133)	Severe (n=168)	p value*
Disposition, n (%)						0.04
Home	154 (32.1)	44 (67.6)	47 (41.2)	28 (21.1)	35 (20.8)	
Expired	139 (29.0)	4 (6.2)	19 (16.7)	44 (33.1)	72 (42.9)	
IPR	107 (22.3)	10 (15.4)	26 (22.8)	36 (27.1)	35 (20.8)	
Long-term Care/Hospice	80 (16.6)	7 (10.8)	22 (19.3)	25 (18.7)	26 (15.5)	
*p-value statistically significant at p <0.05						

**Table 6:** Length of hospital stay based on admission NIHSS NIHSS: National Institutes of Health Stroke Score; SD: standard deviation

Variable	n	Mean ± SD (days)	p value*
Stroke severity			0.09
Mild (NIHSS score 1-4)	65	5.2 ± 2.8	
Moderate (NIHSS score 5-15)	114	9.7 ± 7.5	
Moderate to severe (NIHSS score 16-20)	133	11.5 ± 7.9	
Severe (NIHSS score 21-46)	168	10.3 ± 8.7	
*p-value statistically significant at p <0.05			

**Table 7:** Thrombolysis in cerebral infarction (TICI) score by mode of anesthesia

Variable	Total (N=480)	General Anesthesia (n=257)	Conscious Sedation (n=223)	p value*
TICI Score, n (%)				0.37
0	34 (7.1)	19 (7.4)	15 (6.7)	
1	25 (5.2)	15 (5.8)	10 (4.5)	
2a	59 (12.3)	34 (13.2)	25 (11.2)	
2b	191 (39.8)	98 (38.2)	93 (41.7)	
3	171 (35.6)	91 (35.4)	80 (35.9)	
*p-value statistically significant at p <0.05				

## Discussion

Endovascular therapy in conjunction with aggressive medical management has now become the standard therapy for AIS. With increasing numbers of endovascular procedures taking place, over the last decade there has been mounting literature regarding the use of general anesthesia (GA) versus conscious sedation (CS) during intra-arterial thrombectomy (IAT). Earlier in the decade, retrospective studies and systematic reviews suggested that the use of general anesthesia was fraught with more risk than benefit. Brinjikji et al. completed a systematic review and meta-analysis ultimately analyzing nine studies with a total of 1,956 patients [25]. They reported higher odds of death and respiratory complications in patients undergoing general anesthesia compared to conscious sedation. Proposed theories surrounding the disadvantages of using GA have included delays in revascularization leading to increased neuronal death, increased incidence of hypocapnia in intubated patients leading to arterial vasoconstriction and larger infarct area, and hypotension during induction of anesthesia leading to impaired blood flow through collateral arteries [14,18]. In addition, some authors propose prolonged intubation leading to worsened outcomes in GA patients as well as potential neurotoxicity of the anesthetic agent itself [10].

On the contrary, navigation of microcatheters and devices can be undertaken with greater safety and efficacy in a motionless patient, with dominant hemisphere AIS patients having greater difficulty with understanding instructions due to aphasia that may be a result of their infarction. McDonagh et al. distributed an online survey to 68 active members of the Society of Vascular and Interventional Neurology [26]. The respondents were interventional neurologists in their first 5 years of practice and results showed a preference for GA due to elimination of movement, improved procedural efficacy, and procedural safety. Emiru et al. utilized a different survey approach in which 10 cases were selected at random of patients undergoing IAT for AIS [27]. Major reasons cited for intubation included high initial NIHSS, decreased level of consciousness, increased work of breathing or desaturation, and aphasia limiting the ability to follow commands. The HERMES collaborators, Campbell et. al, published a meta-analysis of patients undergoing general anesthesia for thrombectomy versus thrombectomy with conscious sedation versus standard care in the Lancet Neurology in 2018 [28]. This meta-analysis showed significantly better outcomes for patients who did not receive general anesthesia compared to the general anesthesia cohort. They concluded that general anesthesia should be avoided whenever possible [28].

As evidenced by the vast yet conflicting literature above, there remains a debate regarding the optimal anesthesia modality for intra-arterial thrombectomy. Therefore, we sought to evaluate our own experience at The University of Alabama at Birmingham (UAB) hospital, which is a high volume, Level 1 Stroke Center with a dedicated stroke Neurology and neurointerventionalist team. Our results echo those of the SIESTA and AnStroke trials in that we demonstrated non-inferiority of GA compared to CS. Specifically, there was no significant difference in thrombectomy procedure times, intensive care or hospital length of stay, mean NIHSS improvement from admission to discharge, thrombectomy success, nor discharge disposition between both anesthesia groups. Admission NIHSS was found to be significantly associated with discharge disposition and trended towards significance in its association with hospital LOS.

Our study presents results from a large, continuous patient population; however, it is limited by a single institution, retrospective design. As such, this may limit the generalizability of our findings to those of smaller centers located outside of the Southeast United States with a different patient population. In addition, biases inherent to retrospective studies, such as selection bias, may limit this study as well. Our preliminary data analysis was severely limited by a large imbalance between the GA and CS groups. However, since late 2017, our institution has adopted a policy of using general anesthesia for all thrombectomy cases. This policy change has allowed us to analyze and present more balanced anesthesia groups.

## Conclusions

The results of our study contrast those of previous retrospective analyses, which suggested general anesthesia was fraught with more complications compared to conscious sedation during intra-arterial thrombectomy for acute ischemic stroke. Our findings instead echo those of two previous randomized controlled trials showing non-inferiority when comparing general anesthesia to conscious sedation. In addition, our neurointerventional faculty anecdotally feel that the standard use of general anesthesia in thrombectomy cases also allows for a more controlled workflow which is critical in any emergency procedure.
